# Identification of *Influenza A*/H7N9 Virus Infection-Related Human Genes Based on Shortest Paths in a Virus-Human Protein Interaction Network

**DOI:** 10.1155/2014/239462

**Published:** 2014-05-18

**Authors:** Ning Zhang, Min Jiang, Tao Huang, Yu-Dong Cai

**Affiliations:** ^1^Department of Biomedical Engineering, Tianjin University, Tianjin Key Lab of BME Measurement, Tianjin 300072, China; ^2^State Key Laboratory of Medical Genomics, Institute of Health Sciences, Shanghai Jiaotong University School of Medicine and Shanghai Institutes for Biological Sciences, Chinese Academy of Sciences, Shanghai 200025, China; ^3^Department of Genetics and Genomic Sciences, Mount Sinai School of Medicine, New York City, NY, USA; ^4^Institute of Systems Biology, Shanghai University, 99 Shangda Road, Shanghai 200444, China

## Abstract

The recently emerging *Influenza A*/H7N9 virus is reported to be able to infect humans and cause mortality. However, viral and host factors associated with the infection are poorly understood. It is suggested by the “guilt by association” rule that interacting proteins share the same or similar functions and hence may be involved in the same pathway. In this study, we developed a computational method to identify *Influenza A*/H7N9 virus infection-related human genes based on this rule from the shortest paths in a virus-human protein interaction network. Finally, we screened out the most significant 20 human genes, which could be the potential infection related genes, providing guidelines for further experimental validation. Analysis of the 20 genes showed that they were enriched in protein binding, saccharide or polysaccharide metabolism related pathways and oxidative phosphorylation pathways. We also compared the results with those from human rhinovirus (HRV) and respiratory syncytial virus (RSV) by the same method. It was indicated that saccharide or polysaccharide metabolism related pathways might be especially associated with the H7N9 infection. These results could shed some light on the understanding of the virus infection mechanism, providing basis for future experimental biology studies and for the development of effective strategies for H7N9 clinical therapies.

## 1. Introduction

Influenza is one of the most dangerous contagions worldwide and is still a serious global health threat. In the spring of 2013, a novel* Influenza A* virus subtype H7N9 (A/H7N9) broke out in China and quickly spread to other countries [1–3]. As of 11 August 2013, 136 human infections had been laboratory-confirmed, with 44 deaths.

The* Influenza A* viruses (IAVs) are classified into subtypes according to a combination of 16 hemagglutinin (HA: H1–H16) and 9 neuraminidase (NA: N1–N9) surface antigens [4]. Genomic signature and protein sequence analyses revealed that the genes of this A/H7N9 virus were of avian origin [5–7]. The six internal genes were derived from the avian* Influenza A*/H9N2 strain, whereas the haemagglutinin (HA) and neuraminidase (NA) gene segments were from viruses of domestic duck or wild birds [2, 3, 8].

Generally, most avian influenza viruses (e.g., subtypes H5N1, H9N2, H7N7, and H7N3) are of low pathogenicity [4], possibly because avian viruses are inefficient at binding to sialic acid receptors located in human upper airways [5]. However, by comparison, the novel reassortant A/H7N9 seems to cross species from poultry to human more easily [5]. The recombinant has mutations in the hemagglutinin protein, which is associated with potentially enhanced ability to bind to human-like receptors. A deletion in the viral neuraminidase stalk may be also responsible for the change in viral tropism to the respiratory tract or for enhanced viral replication. Mammalian adaptation mutations are also observed in the polymerase basic 2 (PB2) gene of the virus [2, 9]. These are thought to be correlated with the increased virulence and the better adaptation to mammals of A/H7N9 than other avian influenza viruses [10].

No vaccine for the prevention of A/H7N9 infections is currently available [11]. Although preventing further spread of the infection is important, new drug and vaccine development are also vitally needed for the antiviral treatment. However, viral and host factors associated with the infection of this reassortant are poorly understood [5], which is an obstacle to fight against H7N9. The difficulty is increased by the unusual characteristics from hallmark mutations in the virus, differing from other avian IAVs. Therefore, it is meaningful to identify H7N9 infection-related human genes, which could be used as biomarkers for early diagnosis and targets for new drug development.

In the present study, we proposed a new method for identifying H7N9 infection-related human genes based on a protein-protein interaction (PPI) network. So far the PPI data have been widely used for gene function predictions. The “guilt by association” rule, which was first proposed by Nabieva et al. [12], suggests that interacting proteins share the same or similar functions and hence may be involved in the same pathway. This assumption can be used to identify disease-related genes from existing protein-protein interaction networks. In our previous studies, based on this assumption, we have identified genes related to other diseases, such as the ones mentioned in [13–15].

Shortest path and betweenness method are widely used to identify and analyze biomarkers on virus-host interaction networks [16–18]. If one protein is on many shortest paths between virus target genes, it has great betweenness and it can disrupt the signal transduction on the network [19, 20]. It was found that proteins with great betweenness usually have similar functions with the original seed genes [13, 21]. In this study, we used this method to identify potential host response genes to the A/H7N9 virus infection.

## 2. Materials and Methods

**Figure 1:**
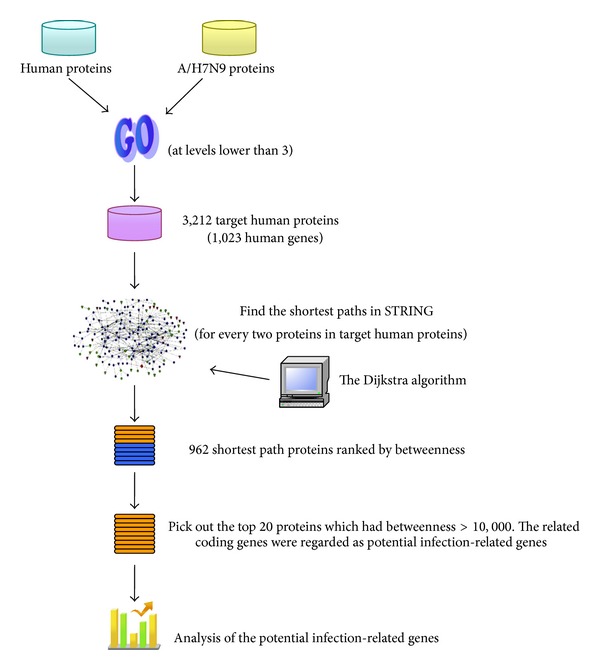
The flowchart of the method developed in this study to identify the* Influenza A*/H7N9 infection-related human genes. Target human proteins interacting with the* Influenza A*/H7N9 virus were obtained based on sharing GO terms. Shortest path proteins were calculated from the shortest paths between every pair of the target human proteins, by searching by the Dijkstra algorithm in the network constructed from STRING. Finally 20 shortest path proteins were screened out with betweenness >10,000, the related genes of which were considered as infection-related human genes.

The overall procedure of our method is illustrated in Figure 1. In the following subsections, details are presented.

### 2.1. Dataset Construction of Target Human Proteins

The course of the* Influenza A*/H7N9 infection can be determined by comprehensive protein-protein interactions (PPIs) between the virus and its host (human). In this study, whether a human protein interacted with virus proteins was determined based on the Gene Ontology (GO) database. The Gene Ontology (GO) terms provide information about the biological process, molecular function, and cellular component of a specific protein. A human protein and a protein of H7N9 having at least 1 sharing GO term were assumed to interact with each other and the human protein was called target human protein. Since protein pairs sharing generic GO terms should be ignored, in this study, only GO terms at levels below 3 were considered. That is to say, we excluded the root GO terms (“GO:0008150: biological_process”, “GO:0005575: cellular_component”, “GO:0003674: molecular_function”), their children, and the children of their children terms. Based on this rule, we constructed a dataset of target human proteins. The detailed description of the procedure was presented below.

All protein sequences of the* Influenza A*/H7N9 virus were downloaded from NCBI protein database (http://www.ncbi.nlm.nih.gov/). After removing those with sequence identities >40%, only 11 proteins were left and were listed in Supporting Information S1 available online at http://dx.doi.org/10.1155/2014/239462. The Gene Ontology (GO) terms at levels below 3 of the 11 proteins were mapped by InterProScan (http://www.ebi.ac.uk/Tools/pfa/iprscan/) [22]. All human proteins and their Protein-GO term mappings were obtained from biomart in ENSEMBL (http://asia.ensembl.org/biomart/martview/).

Based on the rule of sharing GO terms, 3,212 target human proteins (coded by 1,023 human genes) were picked out, each of which interacted with at least 1 H7N9 virus protein. These virus-human protein pairs were provided in Supporting Information S2, together with the sharing GO terms for each pair. And we summarized the 3,212 target human proteins with their 1,023 related coding genes in Supporting Information S3.

### 2.2. PPI Data from STRING

STRING (Search Tool for the Retrieval of Interacting Genes) (http://string.embl.de/) [23] is an online database resource which compiles both experimental and predicted protein-protein interactions with a confidence score to quantify each interaction confidence. A weighted PPI network can be retrieved from STRING, in which proteins in the network are represented as nodes, while interactions between proteins are given as edges marked with confidence scores if they are in interaction with each other. Interacting proteins with high confidence scores in such a PPI network are more likely to share similar biological functions than noninteractive ones [23–25]. This is because the protein and its interactive neighbours may form a protein complex performing a particular function or may be involved in the same pathway.

We constructed a graph *G* with the PPI data from STRING (version 9.0). In such a graph, proteins were represented as nodes; however, the weight of each interaction edge was assigned a *d* value rather than a confidence score (*s*). The *d* value was derived from the confidence score *s* according to the equation *d* = 1000 × (1 − *s*). Thus, the *d* value can be considered as representing protein distances to each other: the smaller the distance, the higher the interaction confidence score and the more similar the functions they have.

In this study, we analyzed in such a graph every two protein interactions in the target human protein dataset.

### 2.3. Shortest Path Tracing

The Dijkstra algorithm [26] were used to find the shortest paths in the graph *G* between every two proteins in the target human protein dataset, that is, the shortest paths between each of the 3,212 proteins to all the other 3,211 proteins in the graph. The Dijkstra algorithm was implemented with R package “igraph” [27] (no parameters needed to be set in this algorithm).

Then we get all proteins existing on the shortest paths (962 proteins, called Shortest Path Proteins) and ranked these proteins according to their betweenness. Results can be found in Supporting Information S4. The top 20 proteins (20 genes) with betweenness over 10,000 were picked out and the 20 corresponding coding genes were regarded as potential H7N9 infection-related human genes.

### 2.4. KEGG Pathway Enrichment Analysis

The functional annotation tool DAVID [28] was used for KEGG pathway enrichment analysis (all parameters were selected as default). The enrichment *P* value was corrected to control family-wide false discovery rate under a certain rate (e.g., ≤0.05) with the Benjamin multiple testing correction method [29]. All human protein-coding genes were regarded as background during the enrichment analysis.

### 2.5. Comparison with Another Two Species of Viruses

To further understand the* Influenza A*/H7N9-human interaction, we compared the results of the potential H7N9 infection-related human genes obtained above with those identified from another two species of viruses: human rhinovirus (HRV) and respiratory syncytial virus (RSV), which are also causing human acute respiratory infections. The same procedure of our method presented above was performed on the two species of viruses as that on H7N9.

All protein sequences of HRV and RSV viruses were downloaded from NCBI protein database (http://www.ncbi.nlm.nih.gov/). After removing those with sequence identities >40%, the proteins left were listed in Supporting Information S5, S6, respectively. The virus-human protein pairs were also provided in Supporting Information S2, and the target human proteins with their coding genes were also summarized in Supporting Information S3. 1,904 and 9,846 shortest path proteins were obtained from HRV and RSV virus, respectively, after computing shortest paths, given also in Supporting Information S4. The numbers of proteins and genes for the three species of viruses at each step were summarized in Table 1.

We selected betweenness threshold as 10,000 for the shortest path proteins of H7N9. However, the threshold should be different for the other two species of viruses since the numbers of target human proteins were different. We standardized the betweenness threshold for HRV and RSV viruses on that for H7N9 virus in this study.

Shortest paths were computed on every two proteins in target human protein dataset. Denoting the number of target human proteins as *N*, the number of shortest paths was *C*_*N*_^2^. The average threshold *w* was calculated on H7N9 as

(1) $$\begin{array}{c} w=\frac{\text{10,000}}{C_{N_{\text{H}7\text{N}9}}^{2}}=\frac{\text{10,000}}{C_{3212}^{2}}=0.001939. \end{array}$$

Then the betweenness threshold for HRV and RSV was determined by *wC*_*N*_HRV__^2^ = *wC*_6985_^2^ = 47,299 and *wC*_*N*_RSV__^2^ = *wC*_36273_^2^ = 1,275,672, respectively. Therefore, the top 11 proteins (11 genes) were picked out for HRV (betweenness > 47,299) while the top 44 proteins (44 genes) were picked out for RSV (betweenness > 1,275,672) from the lists in Supporting Information S4, respectively. And the corresponding 11 and 44 coding genes were regarded as potential infection-related human genes for HRV and RSV virus, respectively. The betweenness threshold and the numbers of proteins picked out were also summarized in Table 1.

## 3. Results and Discussion

### 3.1. Sharing GO Terms between H7N9 Proteins and Human Proteins

**Figure 2:**
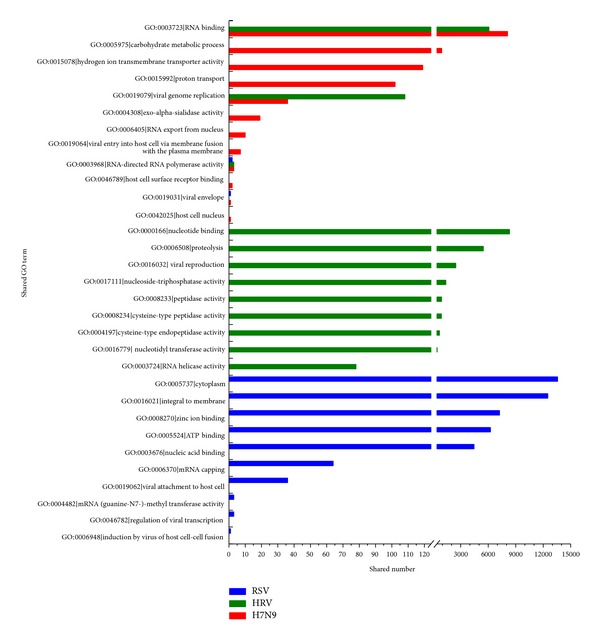
Statistical analysis of sharing GO terms between virus proteins and human proteins. All sharing GO terms and their descriptions between virus and human proteins were listed on the *Y*-axis. Histogram of sharing numbers showed the instances of each term used as a sharing term. The horizontal axis was truncated from 125 to 400.

H7N9 and human proteins with at least 1 sharing GO term were considered as interacting with each other. 3,212 target human proteins were found as interacting with H7N9 proteins based on this rule. The same procedure was performed on the other two species of viruses for comparison: HRV and RSV. Types of the sharing GO terms and the share numbers of the terms could give information about the interaction between the virus and its host. Thus, a statistical analysis was made on the sharing GO terms from Supporting Information S2 for each species of virus, respectively. Results were depicted in Figure 2.

From Figure 2, it can be seen that the sharing GO terms and their numbers were apparently different between the three species of viruses, indicating specific properties and different interactions with host during infections.

For H7N9, the term “GO:0003723∣RNA binding” accounted for the most, indicating important roles of RNA binding proteins in the PPI interactions between H7N9 and human, which was consistent with the observations in* Influenza A* viruses in the literature [30–32]. As shown in Figure 2, H7N9 and HRV both fell into the significant term “GO:0003723∣RNA binding,” indicating that RNA binding was essential between virus-human proteins during the infection of the two viruses. However, RSV was not presented in such a term. It was possibly suggested that H7N9 and HRV had such a specificity that could be different from RSV, although all the three are RNA viruses. Several other GO terms indicated specific and important virus-human protein interactions for H7N9 infection, such as “GO:0005975∣carbohydrate metabolic process,” “GO:0015078∣hydrogen ion transmembrane transporter activity,” and “GO:0015992∣proton transport.”

Nevertheless, 3 terms of H7N9 were the same as those of HRV (“GO:0003723∣RNA binding,” “GO:0019079∣viral genome replication,” and “GO:0003968∣RNA-directed RNA polymerase activity”), and 2 terms as RSV (“GO:0003968∣RNA-directed RNA polymerase activity,” “GO:0019031∣viral envelope”), indicating similar processes of the infections between the three viruses.

### 3.2. Potential H7N9 Infection-Related Genes

**Figure 3:**
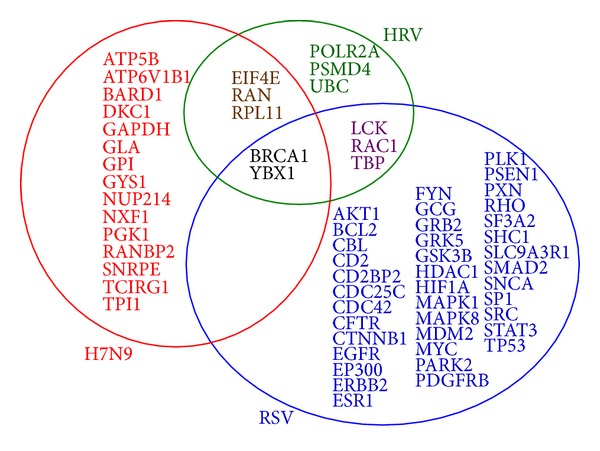
The potential virus infection-related human genes found based on our method for the three species of viruses. 20, 11, and 44 potential infection-related human genes were found for H7N9, HRV, and RSV, respectively. There were 5 sharing genes between those for H7N9 and HRV, and 2 sharing genes between H7N9 and RSV. Other human genes related were not all the same, indicating specific properties or particular characteristics between the infections of the three species of viruses.

The shortest paths were calculated between each pair of the 3,212 proteins. All proteins were picked out with their betweenness from the shortest paths, given in Supporting Information S4. We selected the top 20 proteins with betweenness over 10,000 and ranked them according to their betweenness. The related coding genes of the 20 proteins were also retrieved accordingly (20 genes). These were shown in Table 2. The 20 genes were regarded as potential H7N9 infection-related human genes in this study. Results of potential infection-related human genes for HRV and RSV were also listed in Table 2 by the same method as that for H7N9 for comparison. Note that the proteins (genes) listed in Table 2 were all human proteins (genes), not virus. Potential human genes found for the three viruses were also depicted in Figure 3. It was clearly seen from Figure 3 that the potential human genes found were remarkably different in H7N9 infection as compared with those in HRV and RSV, although several sharing genes existed. Thus, these 20 human genes could be closely related to the H7N9 infections. Our further analysis was based on these 20 genes.

The 20 human genes were submitted to the CCSB interactome database to analyze their interactions with viruses (http://interactome.dfci.harvard.edu/V_hostome/). Among them, proteins encoded by RANBP2 and GYS1were found to be related to EBV or HPV proteins, such as EBV-BVLF1, EBV-BGLF3, and HPV8-E6. These proteins could also have some relationship with H7N9 infections.

Among the 20 genes, some, such as GAPDH and NXF1, had been well documented to be relevant to H7N9 infections. However, there were also other genes with rare previous association with H7N9 infections reported or that had been only poorly characterized, such as PGK1, GYS1, YBX1, and NUP214.

GAPDH (glyceraldehyde-3-phosphate dehydrogenase) is a housekeeping gene in carbohydrate metabolism. This finding was consistent with the general agreement that GAPDH is an important gene and is widely used in the studies of host gene response to virus infections, including influenza virus infections [33–35].

NXF1 (nuclear export factor 1) is one member of a family of nuclear RNA export factor genes. It was reported that viral mRNAs of* Influenza A* virus were transported to the cytoplasm by the NXF1 pathway for translation of viral proteins [36]. Not surprisingly, the H7N9 virus exploited the same pathway.

YBX1 (Y box binding protein 1) has been found to be an interacting partner of genomic RNA of Hepatitis C Virus, which negatively regulates the equilibrium between viral translation/replication and particle production [37]. NUP214 (nucleoporin 214 kDa) encodes one of nucleoporins composing the nuclear pore complex (NPC), which forms a gateway regulating the flow of macromolecules between nucleus and cytoplasm. Many viruses have been reported to require these mechanisms to deliver their genomes into the host cell nucleus for replication, such as human immunodeficiency virus (HIV) [38], encephalomyocarditis virus [39], and herpes simplex virus [40]. However, reports on NUP214, YBX1 related to* Influenza A* viruses, were sparse.

Cancer-related genes were also included. BRCA1 (breast cancer 1) encodes a nuclear phosphoprotein that plays a role in maintaining genomic stability, and it also acts as a tumor suppressor. BARD1 (BRCA1 associated RING domain 1) encodes a protein which interacts with the N-terminal region of BRCA1, regulating cell growth and the products of tumor suppressor genes, and may be related to breast or ovarian cancer.

Interestingly, more genes were involved in energy pathways containing glycolysis and gluconeogenesis, such as GPI (glucose-6-phosphate isomerase), PGK1 (phosphoglycerate kinase 1), and TPI1 (triosephosphate isomerase 1). In addition, GYS1 (glycogen synthase 1) encodes a protein catalyzing the addition of glucose monomers to the growing glycogen molecule in starch and sucrose metabolism. GLA (galactosidase) encodes a glycoprotein that hydrolyses the terminal alpha-galactosyl moieties from glycolipids and glycoproteins. Therefore, it was suggested that the H7N9 infection could be probably linked to saccharide or polysaccharide metabolism related pathways. Central metabolism could be strongly affected by virus infections [41]. Janke et al. [42] also found changes in metabolism in cells infected by* Influenza A*/H1N1 virus, suggesting that fatty acid synthesis might play a crucial role for the virus replication as they acquired lipid.

ATP6V1B1 (ATPase, H+ transporting, lysosomal 56/58 kDa, V1 subunit B1) and ATP5B (ATP synthase, H+ transporting, mitochondrial F1 complex, beta polypeptide) were involved in ATP synthase and hydrolysis.

From Table 2, it also can be seen that although several genes (PGK1, DKC1, was GLA) were located on Chromosome X, none on Chromosome Y was found in this study. Although earlier findings reported that H7N9 infections preferentially occurred in males, it was suggested from our findings that it may not be so significant. This was also consistent with results of Chen et al.'s work [43], in which they indicated that it did not show any statistically significant differences in clinical outcomes between genders from their logistic regression analysis.

### 3.3. GO Enrichment Analysis of H7N9 Infection-Related Genes

We performed GO enrichment analysis on these 20 genes. The 20 proteins encoded by the genes were mapped to GO terms on the levels below 3 from Gene Ontology. Totally 504 GO terms were obtained. GO enrichment analysis was performed on these terms. The GO terms and the number of proteins related to each GO term were shown in Table 3. The same procedure was performed on the other two species of viruses for comparison, with results shown in Table 3. Both commonness and differences of GO term enrichment between the three species of viruses existing as described in Table 3.

Form Table 3, it can be seen that 15 out of the 20 H7N9, all the 11 HRV, and 42 out of the 44 RSV infection-related proteins were involved in protein binding (GO:0005515). Protein binding played important roles in both virus infection and host immune responses [44]. This could partially explain why the novel reassortant had more enhanced ability to bind to human receptors than other avian influenza viruses [2, 10]. The recombinant proteins could also induce immune responses via protein interactions [45]. Once the host immune system activated, patients would have severe symptoms, such as cough, sputum, fever, and shortness of breath. Many related proteins of the three viruses fell into GO terms “GO:0005829 cytosol” and “GO:0005737 cytoplasm,” since all the three viruses are RNA viruses and replication of RNA viruses usually takes place in cytoplasm.

These were commonness. However, differences or specific characteristics still exist in H7N9-related proteins from those of other two viruses.

Nine of these proteins were enriched in “GO:0044281 small molecule metabolic process” (45.00%) for H7N9, whereas only 1 (9.09%) and 2 (4.55%) proteins were enriched in this term for HRV and RSV, respectively. Furthermore, still many related proteins of H7N9 enriched in “GO:0005975 carbohydrate metabolic process,” “GO:0006006 glucose metabolic process,” “GO:0006094 gluconeogenesis,” and “GO:0006096 glycolysis,” differing from those cases of HRV or RSV. These specific enrichment of GO terms indicated that the H7N9 infection could be especially relevant with human saccharide or polysaccharide metabolism-related pathways.

For H7N9, 3 proteins fell into the term “GO:0015991 ATP hydrolysis coupled proton transport” and 3 proteins into “GO:0015992 proton transport,” but it was not the case for HRV or RSV. Proteins involved in “GO:0005215 transporter activity” and “GO:0055085 transmembrane transport” were also different between the H7N9 infections and the other two viruses.

### 3.4. KEGG Pathway Enrichment Analysis

KEGG pathway enrichment analysis was also performed on the 20 genes. The KEGG pathway terms and the number of proteins belonging to each pathway term were shown in Table 4.

Only 3 pathways were retrieved. However, all the 3 pathways were specially related to H7N9; that is, none of the 3 pathways appeared in the KEGG results of the other two viruses (data not shown of the KEGG results for the other two viruses).

Form Table 4, it can be seen that 2 out of the 3 pathways were saccharide or polysaccharide metabolism-related pathways (“Glycolysis/Gluconeogenesis” and “Starch and sucrose metabolism”), suggesting that these types of pathways could play pivotal roles in the H7N9 infections. Another pathway involved was “oxidative phosphorylation.” This pathway could also be important, but it may not so as the former two, since genes involved in this pathway (ATP5B, ATP6V1B1, and TCIRG1) were ranked at the bottom in the gene list in Table 2 according to betweenness.

## 4. Conclusion

In this study, we developed a computational method to identify* Influenza A*/H7N9 infection-related human genes based on the shortest paths in a PPI network. Finally, 20 human genes were screened out which could be the most significant, providing guidelines for further experimental validation. Among the genes, several ones such as PGK1, GYS1, YBX1, and NUP214 were previously reported with rare association with influenza virus infections or had been only poorly characterized in the literature. Most of the 20 genes were enriched in protein binding, saccharide, or polysaccharide metabolism-related pathways and oxidative phosphorylation pathways, compared to the other two viruses HRV and RSV, suggesting direct or indirect relationship with the formation or development of the infection. These candidate genes may provide clues for further researches and experimental validations. Results from this study may shed some light on the understanding of the virus infection mechanism, providing new references for researches into the disease and for new strategies for antivirals, such as new drug and vaccine development.

## Supplementary Material

Supporting Information S1: Sequences of the 11 non-homologous influenza A/H7N9 virus proteins downloaded from NCBI protein database, with pairwise sequence identity less than 40%.
Supporting Information S2: The interacting influenza A/H7N9 virus-human protein pairs with their sharing GO terms. The interacting HRV-human and RSV-human protein pairs were also included respectively.
Supporting Information S3: The 3,212 Target Human Proteins interacted with influenza A/H7N9 virus proteins, found based on sharing GO terms. The Target Human Proteins interacted with HRV and RSV viruses were also included respectively.
Supporting Information S4: The 962 shortest path human proteins picked out on shortest paths for influenza A/H7N9 virus. The shortest path human proteins for HRV and RSV viruses were also included respectively.
Supporting Information S5: Sequences of the 4 non-homologous Human Rhinovirus (HRV) proteins downloaded from NCBI protein database, with pairwise sequence identity less than 40%.
Supporting Information S6: Sequences of the 22 non-homologous Respiratory Syncytical Virus (RSV) proteins downloaded from NCBI protein database, with pairwise sequence identity less than 40%.

## Figures and Tables

**Table 1:** Number of proteins/genes in our datasets of the three species of viruses: *Influenza A*/H7N9 virus, human rhinovirus (HRV), and respiratory syncytial virus (RSV).

Virus	Virus proteins (sequence identity <40%)	Virus-human protein pairs	Target human proteins (coding genes)	Shortest path proteins	Betweenness threshold	Potential infection-related proteins (coding genes)
H7N9	11	9,313	3,212 (1,023)	962	>10,000	20 (20)
HRV	4	20,955	6,985 (2,028)	1,904	>47,299	11 (11)
RSV	22	40,499	36,273 (11,036)	9,846	>1,275,672	44 (44)

**Table 2:** The infection-related human proteins and their related coding genes for the three species of viruses calculated from shortest paths in a PPI network.

Infected by virus	Protein	Gene	Chromosome	Betweenness
*Influenza A*/H7N9	ENSP00000361626	YBX1	1	58376
	ENSP00000363676	RPL11	1	49155
	ENSP00000396127	RAN	12	49036
	ENSP00000362413	PGK1	X	26743
	ENSP00000229239	GAPDH	12	25345
	ENSP00000294172	NXF1	11	23550
	ENSP00000352400	NUP214	9	21849
	ENSP00000280892	EIF4E	4	20883
	ENSP00000379933	TPI1	12	19217
	ENSP00000348877	GPI	19	18885
	ENSP00000317904	GYS1	19	18349
	ENSP00000350283	BRCA1	17	16827
	ENSP00000283195	RANBP2	2	16823
	ENSP00000400591	SNRPE	1	15796
	ENSP00000265686	TCIRG1	11	14465
	ENSP00000358563	DKC1	X	13535
	ENSP00000234396	ATP6V1B1	2	13432
	ENSP00000218516	GLA	X	12471
	ENSP00000262030	ATP5B	12	11891
	ENSP00000260947	BARD1	2	11297

Human Rhinovirus (HRV)	ENSP00000344818	UBC	12	330154
	ENSP00000363676	RPL11	1	154993
	ENSP00000361626	YBX1	1	136548
	ENSP00000357879	PSMD4	1	121991
	ENSP00000337825	LCK	1	117195
	ENSP00000396127	RAN	12	116059
	ENSP00000348461	RAC1	7	111632
	ENSP00000230354	TBP	6	100485
	ENSP00000350283	BRCA1	17	65076
	ENSP00000314949	POLR2A	17	54470
	ENSP00000280892	EIF4E	4	50359

Respiratory syncytial virus (RSV)	ENSP00000269305	TP53	17	15809765
	ENSP00000344456	CTNNB1	3	5756301
	ENSP00000263253	EP300	22	5694027
	ENSP00000339007	GRB2	17	5591895
	ENSP00000275493	EGFR	7	5245421
	ENSP00000270202	AKT1	14	4663263
	ENSP00000264657	STAT3	17	4180564
	ENSP00000350941	SRC	20	3180369
	ENSP00000348461	RAC1	7	3066312
	ENSP00000221494	SF3A2	19	2994393
	ENSP00000417281	MDM2	12	2686189
	ENSP00000338345	SNCA	4	2647616
	ENSP00000206249	ESR1	6	2643164
	ENSP00000296271	RHO	3	2573058
	ENSP00000329623	BCL2	18	2541856
	ENSP00000376609	GRK5	10	2364221
	ENSP00000337825	LCK	1	2306232
	ENSP00000314458	CDC42	1	2174421
	ENSP00000262613	SLC9A3R1	17	2097178
	ENSP00000355865	PARK2	6	2033100
	ENSP00000264033	CBL	11	1930392
	ENSP00000269571	ERBB2	17	1922027
	ENSP00000338018	HIF1A	14	1915325
	ENSP00000324806	GSK3B	3	1910676
	ENSP00000215832	MAPK1	22	1831541
	ENSP00000358490	CD2	1	1751073
	ENSP00000262160	SMAD2	18	1727787
	ENSP00000304903	CD2BP2	16	1714523
	ENSP00000362649	HDAC1	1	1703720
	ENSP00000353483	MAPK8	10	1702626
	ENSP00000261799	PDGFRB	5	1679113
	ENSP00000003084	CFTR	7	1662248
	ENSP00000401303	SHC1	1	1548773
	ENSP00000321656	CDC25C	5	1521621
	ENSP00000357656	FYN	6	1503978
	ENSP00000326366	PSEN1	14	1498004
	ENSP00000230354	TBP	6	1458835
	ENSP00000300093	PLK1	16	1444680
	ENSP00000350283	BRCA1	17	1389799
	ENSP00000228307	PXN	12	1358706
	ENSP00000329357	SP1	12	1347630
	ENSP00000361626	YBX1	1	1342956
	ENSP00000387662	GCG	2	1321174
	ENSP00000367207	MYC	8	1284185

**Table 3:** GO term enrichment analysis of the 20 potential H7N9 infection-related human proteins (data not shown of GO terms with number of related proteins below 3) and comparisons with HRV and RSV for these terms.

GO terms	H7N9		HRV		RSV	
	Number of proteins	Percentage accounting for the 20 proteins (%)	Number of proteins*	Percentage accounting for the 11 proteins (%)*	Number of proteins*	Percentage accounting for the 44 proteins (%)*
GO:0005515 protein binding	15	75.00	11	100.00	42	95.45
GO:0005829 cytosol	13	65.00	7	63.64	23	52.27
GO:0005737 cytoplasm	11	55.00	6	54.55	30	68.18
GO:0005634 nucleus	9	45.00	4	36.36	33	75.00
GO:0044281 small molecule metabolic process	9	45.00	1	9.09	2	4.55
GO:0003723 RNA binding	8	40.00	4	36.36	2	4.55
GO:0005975 carbohydrate metabolic process	8	40.00	—	—	—	—
GO:0005654 nucleoplasm	7	35.00	7	63.64	19	43.18
GO:0010467 gene expression	7	35.00	8	72.73	6	13.64
GO:0006006 glucose metabolic process	5	25.00	—	—	2	4.55
GO:0005886 plasma membrane	4	20.00	4	36.36	27	61.36
GO:0005622 intracellular	4	20.00	3	27.27	7	15.91
GO:0005643 nuclear pore	4	20.00	1	9.09	—	—
GO:0016032 viral reproduction	4	20.00	8	72.73	4	9.09
GO:0016070 RNA metabolic process	4	20.00	4	36.36	1	2.27
GO:0006094 gluconeogenesis	4	20.00	—	—	—	—
GO:0006096 glycolysis	4	20.00	—	—	—	—
GO:0055085 transmembrane transport	4	20.00	—	—	1	2.27
GO:0005625 soluble fraction	3	15.00	—	—	5	11.36
GO:0008270 zinc ion binding	3	15.00	2	18.18	11	25.00
GO:0016071 mRNA metabolic process	3	15.00	4	36.36	1	2.27
GO:0006606 protein import into nucleus	3	15.00	1	9.09	1	2.27
GO:0005524 ATP binding	3	15.00	1	9.09	14	31.82
GO:0006406 mRNA export from nucleus	3	15.00	1	9.09	—	—
GO:0008286 insulin receptor signaling pathway	3	15.00	1	9.09	4	9.09
GO:0005215 transporter activity	3	15.00	—	—	—	—
GO:0015991 ATP hydrolysis coupled proton transport	3	15.00	—	—	—	—
GO:0015992 proton transport	3	15.00	—	—	—	—
GO:0019221 cytokine-mediated signaling pathway	3	15.00	2	18.18	1	2.27

*—: no proteins having the GO term was picked out as potential infection-related proteins for the virus.

**Table 4:** KEGG pathway enrichment analysis of the 20 potential H7N9 infection-related human genes.

Terms	Genes	Number of genes belonging to the pathway	Percentage accounting for the 20 genes (%)	Adjusted *P* value (Benjamini)
Glycolysis/Gluconeogenesis	TPI1, GPI, GAPDH, and PGK1	4	20.00	7.0*E* − 3
Oxidative phosphorylation	ATP5B, ATP6V1B1, and TCIRG1	3	15.00	3.3*E* − 1
Starch and sucrose metabolism	GPI, GYS1	2	10.00	5.2*E* − 1
